# Parathyroid imaging with ^18^F-fluorocholine PET/CT as a first-line imaging modality in primary hyperparathyroidism: a retrospective cohort study

**DOI:** 10.1186/s13550-019-0544-3

**Published:** 2019-07-31

**Authors:** Wouter A. M. Broos, Maurits Wondergem, Remco J. J. Knol, Friso M. van der Zant

**Affiliations:** Department of Nuclear Medicine, Northwest Clinics, Wilhelminalaan 12, 1815 JD Alkmaar, The Netherlands

**Keywords:** ^18^F-fluorocholine, PET/CT, Primary hyperparathyroidism, Parathyroid adenoma

## Abstract

**Background:**

^18^F-fluorocholine (FCH) PET/CT is a promising technique for visualizing hyperfunctioning parathyroid glands in hyperparathyroidism. It is still under debate whether to use this technique as a first-line imaging modality or to use it when conventional techniques such as ^99m^Tc-sestamibi scintigraphy or ultrasonography are inconclusive. This study evaluates FCH PET/CT as a first-line modality.

**Methods:**

Patients with primary hyperparathyroidism, referred between June 2015 and December 2018 for FCH PET/CT as a first-line imaging method, were included in this study. Baseline characteristics, clinical data, scan results, and type of treatment were recorded. The rate of correct detection was calculated on a per patient-based and a per lesion-based analysis. The reference standard comprised histopathological results, intraoperative response to parathyroidectomy, and clinical follow-up.

**Results:**

Two hundred and seventy-one patients were included, of which 139 patients underwent parathyroidectomy, 48 were treated with calcimimetics, and 84 patients received further follow-up without active treatment. In the surgically treated group, a single adenoma was suspected in 127 scans, double adenoma in three scans, and one scan showed evidence of three hyperfunctioning glands. In eight scans, no lesions were visualized. A total of 154 parathyroid glands were surgically removed. The rate of correct detection was calculated at 96% and 90%, on a per patient-based and per lesion-based analysis, respectively.

**Conclusion:**

This retrospective study in a large cohort shows high detection rates of FCH PET/CT in primary hyperparathyroidism, which is in accordance to literature. The use of FCH PET/CT as a first-line imaging modality in preoperative planning of parathyroid surgery may therefore be a suitable choice.

## Background

Primary hyperparathyroidism is a relatively common endocrine disorder, which develops as a result of autonomous overproduction of parathyroid hormone (PTH) by parathyroid glands. In most cases, the disease is caused by a single parathyroid adenoma (89%) and less commonly by parathyroid hyperplasia (6%), double parathyroid adenoma (4%), or parathyroid carcinoma (< 1%) [1].

Clinical presentation is heterogeneous. Asymptomatic disease is the most frequent presentation in Western countries where routine laboratory testing is common [2, 3]. Kidney stones occur in 15–20% and a second important clinical feature is bone loss with associated fracture risk [4].

Primary hyperparathyroidism is routinely treated with parathyroidectomy, which still is the only definitive management. In surgical treatment of hyperparathyroidism, precise preoperative localization of hyperfunctioning parathyroid tissue has become increasingly important, due to a shift from bilateral neck exploration to minimally invasive parathyroidectomy in the past decades [5].

The current reference method for localization of hyperfunctioning parathyroid glands is ^99m^Tc-sestamibi scintigraphy, with reported detection rates of 84–88% [6, 7]. In recent years, PET/CT imaging with ^18^F-fluorocholine (FCH) has gained interest for parathyroid imaging. This technique provides higher spatial resolution, shorter scanning time, and less radiation dose and has provided promising results with detection rates exceeding 90% [8, 9].

Whether to use this technique as a first-line imaging modality, or to use it when conventional techniques are inconclusive, is still under discussion. In this study, the use of FCH PET/CT as a first-line modality was evaluated.

## Methods

### Patient selection

Patients who received FCH PET/CT for analysis of primary hyperparathyroidism from June 2015 through December 2018 were included in this study. Primary hyperparathyroidism, according to the American Association of Endocrine Surgeons Guidelines, was defined as the persistent elevation of total serum calcium levels with corresponding elevated or inappropriately normal PTH levels, or high PTH levels and normal serum calcium levels [10]. Patients with familial hypocalciuric hypercalcemia were excluded.

When patients were referred more than once for FCH PET/CT imaging, all scans except for the most recent scan before surgery were excluded. When patients received multiple scans but no surgery, the follow-up scans were excluded. Patients who were on the waiting list for parathyroidectomy were also excluded. All patient data, including baseline characteristics and scan results, were entered into a database. Additional follow-up data were retrieved from the electronic patient records.

### Informed consent

All patients gave written informed consent for use of their anonymized data for scientific purposes. Besides the standard imaging protocol and clinical management, no additional measurements or actions affecting the patient were performed. The study was approved by the institutional research department and performed in accordance with the Declaration of Helsinki. Approval of the local ethical committee for the present study was not necessary since the study does not fall within the scope of the Dutch Medical Research Involving Human Subjects Act (section 1.b WMO, 26th February 1998).

### Scan acquisition

PET/CT images were acquired on a Siemens Biograph-16 TruePoint PET/CT camera (Siemens Healthcare, Knoxville, TN, USA). Dual-time-point images were acquired at 5 min and 60 min after intravenous injection of approximately 150 MBq FCH, ranging from the temporomandibular joint to the diaphragm. Images were acquired at 480 s per bed position with matrix size 256 × 256 and low-dose CT for attenuation correction using a tube current of 40–80 mAs at 100–120 kV with CARE Dose 4D dose modulation, collimation of 24 × 1.2 mm and a pitch of 0.95. PET images were reconstructed with an iterative 3D method using 5 iterations, 8 subsets, and a Gaussian filter. Interpretation of the images and quantitative measurements were done with dedicated software (Syngo.via, Siemens Medical Solutions, Malvern, PA, USA). Patient preparation consisted of hydration with 1 L water and, if applicable, discontinuation of colchicine 48 h prior to FCH administration. Discontinuation of calcimimetic drugs or other medications was not required.

### Image interpretation

**Fig. 1:**
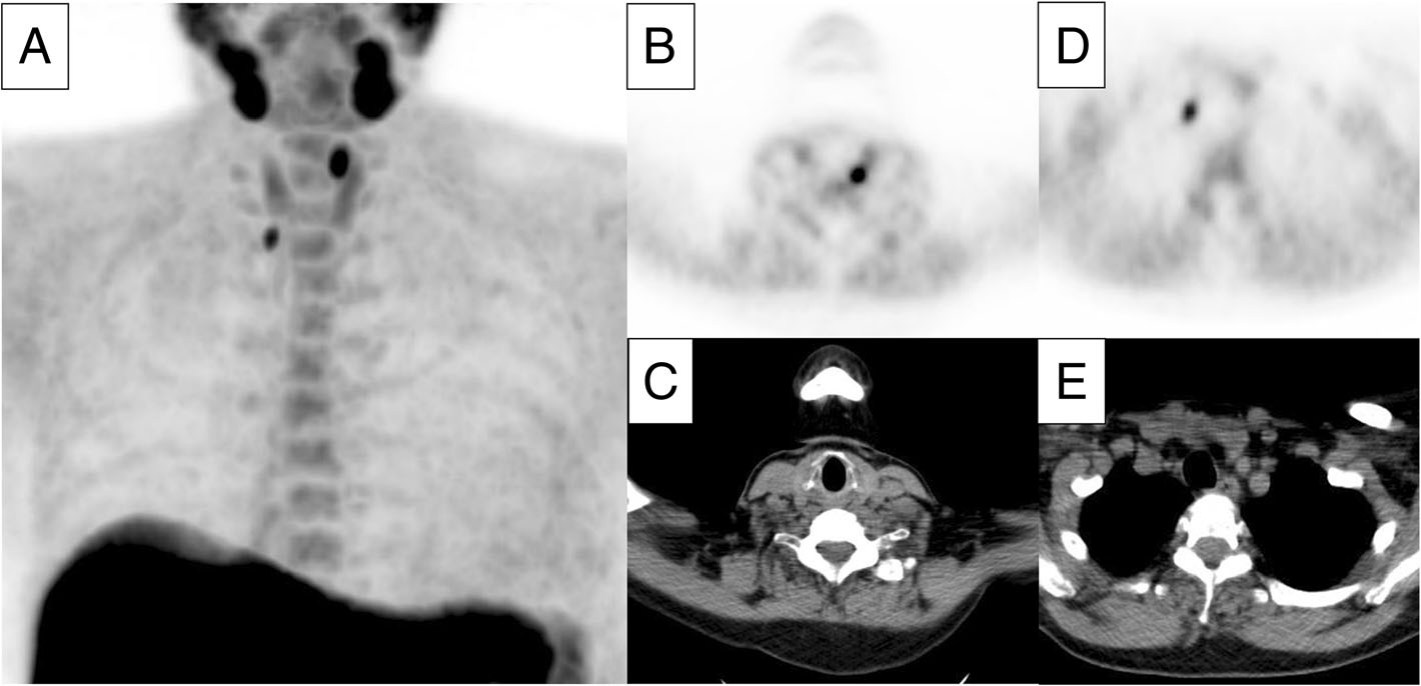
Example of a positive ^18^F-fluorocholine PET/CT representing a double parathyroid adenoma. Serum calcium level in this patient was 2.61 mmol/L with a parathyroid hormone level of 27.7 pmol/L. The maximum intensity projection image (**a**) shows two foci of intense choline uptake in the neck and physiological uptake in the salivary glands, thyroid gland, liver, spleen, and bone marrow. Axial views of PET and CT show foci suspicious for parathyroid adenomas located dorsocranial to the left thyroid lobe (**b**, **c**, dimensions 7 × 5 mm and SUV_max_ of 8.7) and inferior to the right thyroid lobe (**d**, **e**, dimensions 6 × 4 mm and SUV_max_ of 5.6)

Imaging results were prospectively recorded in a database after reading of the scan. Each FCH PET/CT was scored as negative, equivocal, or positive for hyperfunctioning parathyroid glands. In the presence of well-defined focal FCH uptake found in typical locations, the scan was considered positive (Fig. 1). A normal distribution of FCH or atypical tracer distribution with an alternative explanation rather than hyperfunctioning parathyroid tissue defined the scan as negative. Equivocal scans were discussed in a consensus meeting with three nuclear medicine physicians (W.B., F.Z., and M.W.), blinded from other patient data, and scored as positive or negative. The location and number of foci were also entered into the database. For scans positive for parathyroid adenoma, the SUV_max_ of the lesion was measured, if applicable, on both the early and late images.

### Statistical analysis

Histopathological findings in combination with intraoperative results (gland inspection by the surgeon and intraoperative PTH monitoring) and clinical follow-up data were used as the reference standard. The rate of correct detection was calculated as the number of true positives divided by the sum of true positives and false negatives. Detection rates were calculated in a per patient-based and per lesion-based analysis. Pearson’s correlation coefficient was calculated to evaluate the correlation between parameters such as laboratory values, SUV_max_ of the lesions, and adenoma size and weight. Normally distributed continuous data were expressed as mean ± standard deviation (SD) and range. Non-continuous data were expressed as numbers with percentages. The analysis was carried out using the Statistical Package for Social Sciences 20 (IBM SPSS Statistics, Chicago, IL, USA).

## Results

A total of 294 FCH PET/CT scans were performed for analysis of primary hyperparathyroidism during the studied period. Eight scans were excluded because there was a more recent scan available before surgery. Three scans were excluded because these were follow-up scans. Another 12 patients were excluded since they were lost-to-follow-up due to surgery in another hospital, death before surgery, or because patients were still on the waiting list for parathyroidectomy at the time of analysis. A total of 271 patients were included in this study. All FCH PET/CT scans were performed as first-line imaging for localization of hyperfunctioning parathyroid glands as is the standard of care in our institution. Patients were referred from two different hospitals, while imaging and surgery were performed in the same institution.

Patient characteristics and laboratory values (serum calcium, PTH, phosphate, and 24-h urinary calcium levels) are presented in Table 1. Five patients were known with multiple endocrine neoplasia syndrome (three patients with MEN1 and two patients with MEN2a) and 17 patients had lithium associated disease. Ten patients had a history of parathyroid surgery and 7 patients had a history of thyroid surgery. Of the MEN1 patients, one had previous parathyroid surgery. Both MEN2a patients had previous thyroid surgery. Symptomatic disease was present in 146 patients; 51 patients had kidney stones or nephrocalcinosis and 84 patients had osteoporosis or insufficiency fractures. Other reported symptoms, likely to be related to the disease, were musculoskeletal complaints, abdominal pains, nausea, and fatigue.

**Table 1:** Patient characteristics

	All patients *n* = 271		Operated patients *n* = 139		Patients treated with calcimimetics *n* = 48		Patients without active treatment *n* = 84	
Age (years)	62 ± 12 (29–86)*		60 ± 11 (29–81)*		67 ± 11 (43–86)*		64 ± 11 (31–83)*	
Sex (*n*)								
Female	205 (76%)		107 (77%)		34 (71%)		64 (76%)	
Male	66 (24%)		32 (23%)		14 (29%)		20 (24%)	
PTH (pmol/L) (normal range 1.3–6.8 pmol/L)	16.1 ± 11.3 (2.4–121.9)*	*n* = 271	17.9 ± 13.5 (4.5–121.9)*	*n* = 139	16.7 ± 11.2 (4.3–57.1)*	*n* = 48	12.6 ± 5.1 (2.4–26.0)*	*n* = 84
Serum calcium (mmol/L) (normal range 2.10–2.50 mmol/L)	2.66 ± 0.21 (2.11–3.86)*	*n* = 271	2.72 ± 0.21 (2.11–3.86)*	*n* = 139	2.67 ± 0.20 (2.14–3.14)*	*n* = 48	2.56 ± 0.18 (2.16–2.96)*	*n* = 84
Serum phosphate (mmol/L) (normal range 0.7–1.4 mmol/L)	0.9 ± 0.2 (0.3–1.5)*	*n* = 223	0.8 ± 0.2 (0.3–1.2)*	*n* = 120	0.9 ± 0.2 (0.6–1.2)*	*n* = 38	0.9 ± 0.2 (0.5–1.5)*	*n* = 65
24-h urinary calcium (mmol/24 h) (normal range 2.5–7.5 mmol/24 h)	7.2 ± 4.0 (0.1–18.5)*	*n* = 198	8.8 ± 3.9 (0.1–18.5)*	*n* = 99	6.4 ± 3.5 (0.7–14.3)*	*n* = 37	5.6 ± 3.8 (0.5–17.5)*	*n* = 62
SUV_max_ (early images)	6.1 ± 2.9 (1.5–20.5)*	*n* = 197	6.3 ± 2.7 (1.5–12.7)*	*n* = 127	5.9 ± 2.6 (2.7–12.5)*	*n* = 36	5.7 ± 3.8 (2.1–20.5)*	*n* = 34
SUV_max_ (late images)	4.9 ± 2.2 (1.3–13.7)*	*n* = 203	5.0 ± 2.2 (1.3–12.0)*	*n* = 132	4.7 ± 2.4 (1.6–13.7)*	*n* = 37	4.4 ± 2.0 (1.8–9.9)*	*n* = 34

*PTH* parathyroid hormone, *SUV*_*max*_ maximum standardized uptake value

*mean ± SD (range)

Scans were positive for hyperfunctioning parathyroid glands in 202 patients (75%) and negative in 69 patients (25%), of which 16 were initially scored as equivocal (6 positives and 10 negatives after consensus). A total of 213 foci that were suspicious for hyperfunctioning parathyroid glands were detected; 23 foci (11%) were located left superior, 74 (35%) left inferior, 22 (10%) right superior, 85 (40%) right inferior, and nine (4%) glands were ectopically located. Parathyroidectomy was performed in 139 patients (51%), 48 patients (18%) were treated with calcimimetics, and 84 patients (31%) received no active treatment. Of the pharmacologically treated patients, most received 30 mg of Cinacalcet per day (*n* = 36), and the other patients received dosages of 7.5 mg (*n* = 1), 60 mg (*n* = 8), 90 mg (*n* = 1), or 120 mg (*n* = 2) per day. Demographics and laboratory values of each subgroup are listed in Table 1. Mean follow-up was 22 ± 11 months (range 3–43 months).

Ten patients showed evidence of multiple active glands; nine patients with two foci and one patient with three foci. Of the patients with two foci, only three underwent surgery. In two of those patients, the two corresponding parathyroid glands were removed and histopathologically confirmed to be adenomas. In the other patient, only one parathyroid adenoma was found at surgery after which the patient was cured indicating a false-positive finding on the scan. The patient with three foci, a patient with MEN2a syndrome, underwent surgery as well. In this patient, two out of the three foci were confirmed to be adenomas. The other MEN patients had no signs of multiglandular disease on PET/CT.

The subgroup with patients that underwent surgery was further analyzed. Cure was achieved in 136 patients. In two patients, no parathyroid adenoma was found intraoperatively, both with a negative scan, and in one patient, a parathyroid adenoma was removed but elevated PTH persisted during follow-up. In all other patients, intraoperative PTH significantly decreased to normal values or showed at least a 50% reduction and no recurrence occurred during follow-up. In these patients, also serum calcium levels normalized without relapse during follow-up. The type of surgery was minimally invasive in 75% of the procedures, and a standard Kocher incision was performed in the other 25%, in some cases combined with bilateral exploration. A total of 154 parathyroid glands were removed. Fourteen (9%) of the removed glands were located left superior, 57 (37%) left inferior, 15 (10%) right superior, 64 (41%) right inferior, and four (3%) glands were ectopically located. Histopathological results revealed adenoma in 133 of the removed glands (86%) and hyperplasia in five (3%), and in 16 cases (10%), the pathology report confirmed that the removed tissue contained parathyroid gland without further specification. When serum PTH decreased after removal of the glands, these were considered as hyperfunctioning parathyroid glands. The mean weight of the removed glands was 1.06 g (SD 2.43, 95% CI 0.62–1.49), the mean maximum length was 1.60 cm (SD 0.95, 95% CI 1.43–1.76), and the mean volume was 1.74 cm^3^ (SD 4.79, 95% CI 0.81–2.68).

In the subgroup of 139 operated patients, 131 scans were positive for hyperfunctioning parathyroid tissue. These could all be designated as true positives after correlation with surgical and histopathological data. Six of the eight negative scans proved to be false negatives during surgery. This resulted in a calculated detection rate of 96% (95% CI 91–98%), on a per patient-based analysis. In the per lesion-based analysis, 133 of the 154 removed glands were correctly detected on the scans. There were 15 histopathologically proven parathyroid adenomas that were not detected on the scans. Calculated per lesion-based detection rate was therefore 90% (95% CI 84–94%). These results are summarized in Table 2. Detection of hyperfunctioning glands was lower in the non-operatively treated subgroups; 38 out of 48 (79%) in the subgroup treated with calcimimetics, and only 33 out of 84 (39%) in the subgroup receiving no active treatment. Because no reference standard was available in those patients, the rate of correct detection could not be calculated for these subgroups.

**Table 2:** Scan performance

	Total	True positive	False negative	Correct detection rate (95% CI) (%)
Patient-based	139	131	6	96 (91–98)
Lesion-based	154	133	15	90 (84–94)

*CI* confidence interval

The SUV_max_ was measured on 197 of the early images and on 203 of the late images. The mean SUV_max_ on the early images was 6.1 and decreased to a mean SUV_max_ of 4.9 on the late images, which is in line with our previous study on this matter [11]. The uptake values for each subgroup are shown in Table 1. These measurements, along with adenoma sizes and weight which were retrieved from the pathology reports, were correlated with laboratory values by calculating the Pearson’s correlation coefficient (*r*). Weak to very weak correlations were found between SUV and laboratory values (*r* values of 0.12–0.31) and size or weight (*r* values of 0.17–0.38). There was a moderate correlation between the serum calcium level and adenoma weight (*r* = 0.40) and volume (*r* = 0.41), and a strong correlation was found between the PTH level and weight (*r* = 0.69) and volume (*r* = 0.72). Weight and size were only weakly correlated with serum phosphate levels (*r* values of − 0.24 and − 0.25, respectively). No significant *r* could be calculated between 24-h urinary calcium levels and other parameters.

## Discussion

In this study, the performance of FCH PET/CT as a first-line imaging method was investigated. Most studies that have been published on the use of FCH PET/CT in hyperparathyroidism used this scan as a second-line imaging method when conventional imaging methods, such as ^99m^Tc-sestamibi scintigraphy and ultrasonography, were inconclusive, or when previous surgery had failed [12–19]. Fewer studies were published on the performance of FCH PET/CT as a first-line imaging modality, with reported sensitivities ranging between 89% and 100% [20–23]. The results from the present study, with a detection rate of 96% and 90%, on a per patient-based and a per lesion-based respectively, are in line with these earlier studies.

In this study, the detection rate was calculated as the number of true positives divided by the total of true positives and false negatives. The term detection rate was used instead of sensitivity, because of the highly selected patient population which only contained patients with biochemically proven primary hyperparathyroidism. Additionally, the purpose of imaging is not to confirm or exclude the disease, but to detect and localize the diseased parathyroid glands. Frequently, true negative numbers are not available, because acquiring histopathological proof of intraoperatively normal-looking glands is not recommended and in minimally invasive parathyroidectomy, the other parathyroid glands are not inspected [10]. In this study, both the per patient-based as well as the per lesion-based detection rate were calculated. The use of the lesion-based detection rate is most appropriate, because this number represents the correct localization of the gland, which is essential in preoperative planning. The main strength of this study is the large cohort size, including a substantial group of patients that underwent parathyroidectomy. However, 18% of the patients were treated with medication and 31% received further follow-up without active treatment, and for this part of the cohort, no detection rate could be calculated because of the lack of a reference standard. This subjects the analysis of the operated patient group to selection bias and possibly leads to an overestimation of the detection rates, which are, nevertheless, in accordance with the literature. Prospective trials could reduce this selection bias.

Besides the excellent detection rates of FCH PET/CT, there are several other benefits of this modality above conventional scintigraphy. FCH PET/CT has shorter acquisition times and lower radiation dose, and there is no need to stop calcimimetic drugs. PET/CT scanners have become more commonly available, and the tracer is suitable to be shipped due to the relatively long half-life of 110 min. In general, PET/CT is more expensive than conventional scintigraphy; however, costs can be reduced if an on-site cyclotron is available or when a hospital can act as a referral center with frequent use of the technique. Also, the cost of radiopharmaceuticals could be reduced with more sensitive scanners and more sophisticated software. Cost-effectiveness was not evaluated in this study and has not been investigated in earlier studies but will be relevant for the decision whether to use FCH PET/CT as the first-line imaging method instead of conventional scintigraphy.

It is expected that detection rates will improve with new generation PET/CT scanners. Present digital systems have effective sensitivities up to 98.4 counts per second (cps) per kBq, compared to 9 cps/kBq for the analog Siemens TruePoint PET/CT, used in this study. This is especially relevant for the detection of small lesions such as parathyroid glands. Besides FCH PET/CT, another promising imaging technique is contrast-enhanced CT acquired at multiple time points (4D-CT). Although the performance of CT is good, the main disadvantage is the higher radiation dose [24]. A pilot study demonstrated that FCH PET/CT is comparable in performance to 4D-CT [25], a recent study showed that the combination of FCH PET with 4D-CT was of added value [18], and a third study concluded that 4D-CT appears as a confirmatory imaging modality [19]. Furthermore, an interesting development is the use of FCH PET/MRI, with the reduction of radiation dose and increased soft-tissue resolution [16, 26, 27].

In our institution, choline PET/CT is performed as a first-line imaging method in the routine clinical work-up of hyperparathyroid patients. No ^99m^Tc-sestamibi scintigraphy, CT, or MRI was performed before choline PET/CT in these patients. Also, ultrasound is no prerequisite in our institution. Only patients with primary hyperparathyroidism were included in this study; therefore, no conclusions can be drawn on the use of choline PET/CT as a first-line imaging method in secondary of tertiary hyperparathyroidism. The group of patients in this study was heterogeneous, representing the population in our institution. Both symptomatic and asymptomatic patients were represented and patients with typical elevated serum calcium and PTH levels but also with normocalcemic hyperparathyroidism were included.

In the group of patients who underwent surgery, high detection rates were found, and it can be concluded that in this setting, the choline PET/CT is a useful method to guide the surgeon. A noteworthy part of the studied group, however, did not undergo surgery. In this group (treated with medication or without active treatment), the exact value of the choline PET/CT cannot be assessed, since the influence of the scan on further treatment of the patient and clinical outcome is unclear. One possible explanation for the relatively high rate of non-operated patients is that FCH PET/CT is performed rather early in the diagnostic process in our institution. Regularly, the patients are referred for choline PET/CT by the endocrinologist and are referred for surgical intervention at a later time point. However, the general consensus is that imaging of parathyroid glands is performed for preoperative localization in patients for which operation is already indicated [10]. The value of choline PET/CT or other parathyroid imaging techniques to prove or rule out parathyroid disease is unknown.

## Conclusion

This retrospective cohort study shows high detection rates of FCH PET/CT in primary hyperparathyroidism, which is in accordance with literature. The use of FCH PET/CT as a first-line imaging modality in preoperative planning of parathyroid surgery seems an appropriate choice; however, cost-effectiveness analyses are warranted.

## Data Availability

Please contact the author for data requests.

## Abbreviations

4D-CT: Four-dimensional computed tomography
cps: Counts per second
CT: Computed tomography
FCH: ^18^F-fluorocholine
MEN: Multiple endocrine neoplasia
MRI: Magnetic resonance imaging
PET: Positron emission tomography
PTH: Parathyroid hormone
SUV_max_: Maximum standardized uptake value
